# Elementary Treatise on Internal Pathology

**Published:** 1845-04

**Authors:** 


					﻿BIBLIOGRAPHICAL NOTICES.
Traite El&mentaire de, Pathologie Interne. Par MM. A. Hardy
et J. Behier, &c. &c. Tome premier—Pathologie Generale et
Semeiologie. 8vo. pp. 712. Paris, 1844.
Elementary Treatise on Internal Pathology. By MM. A.
Hardy and J. Behier. Vol. first. General Pathology and
Semeiology, &c. &c.
Traits Elementaire et Pratique de Pathologie Interne. Par
A. Grisolle, D. M. P., &c. &c. 8vo. 2 vols.: pp. 812, 912.
Paris, 1844,
				

